# One Health Approach to Leishmaniases: Understanding the Disease Dynamics through Diagnostic Tools

**DOI:** 10.3390/pathogens9100809

**Published:** 2020-10-01

**Authors:** Ahyun Hong, Ricardo Andrade Zampieri, Jeffrey Jon Shaw, Lucile Maria Floeter-Winter, Maria Fernanda Laranjeira-Silva

**Affiliations:** 1 Department of Physiology, Institute of Biosciences, University of São Paulo, São Paulo 05508-090, Brazil; avery.ahyun.hong@ib.usp.br (A.H.); ricardoz@ib.usp.br (R.A.Z.); lucile@ib.usp.br (L.M.F.-W.); 2 Department of Parasitology, Institute of Biomedical Sciences, University of São Paulo, São Paulo 05508-000, Brazil; jeffreyj@usp.br

**Keywords:** *Leishmania*, protozoan parasite, epidemiology, environment, diagnosis

## Abstract

Leishmaniases are zoonotic vector-borne diseases caused by protozoan parasites of the genus *Leishmania* that affect millions of people around the globe. There are various clinical manifestations, ranging from self-healing cutaneous lesions to potentially fatal visceral leishmaniasis, all of which are associated with different *Leishmania* species. Transmission of these parasites is complex due to the varying ecological relationships between human and/or animal reservoir hosts, parasites, and sand fly vectors. Moreover, vector-borne diseases like leishmaniases are intricately linked to environmental changes and socioeconomic risk factors, advocating the importance of the One Health approach to control these diseases. The development of an accurate, fast, and cost-effective diagnostic tool for leishmaniases is a priority, and the implementation of various control measures such as animal sentinel surveillance systems is needed to better detect, prevent, and respond to the (re-)emergence of leishmaniases.

## 1. Introduction

**Figure 1:**
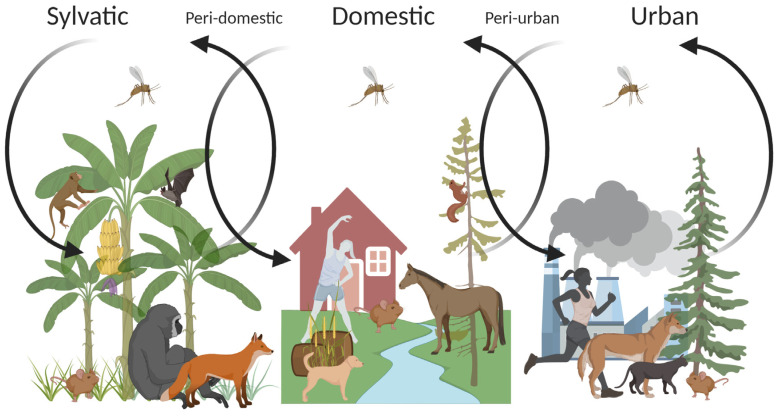
The transmission cycles of zoonotic leishmaniases. Sylvatic leishmaniases can spill over into humans living in proximity to forest foci of transmission, mainly due to deforestation or other factors affecting the ecological balance. As depicted by arrows, sand fly vectors, whose primary forests are their natural breeding sites, adapt to peri-domestic and domestic environments and eventually invade densely populated urban environments (modified from [9]).

Leishmaniases are vector-borne diseases caused by protozoan parasites of the genus *Leishmania* and are transmitted amongst mammalian hosts by phlebotomine sandflies. They are endemic in 98 countries and are estimated to affect over 350 million people around the globe [1,2]. The diseases can be categorized into two types according to the primary reservoir hosts of the human infection: zoonosis and anthroponosis. Zoonosis refers to an infectious disease of animals that can be transmitted to humans, and anthroponosis refers to a naturally occurring infectious disease among humans [3,4]. The majority of the *Leishmania* species are involved in zoonotic transmission. Infected animal reservoir hosts are often introduced into the human population and spillover events result in zoonotic diseases (Figure 1). Twenty-two *Leishmania* species belonging to the subgenera *L. (Leishmania*), *L.* (*Mundinia*), and *L*. (*Viannia*) [5] are found in humans. Among these, just two *L. (L.) donovani* and *L. (L.) tropica* are associated with an anthroponotic cycle (Table 1). However, infections of both species have been reported in livestock animals in Nepal [6,7], and in hyraxes and dogs in the Mediterranean Basin and several countries in Africa [8].

Leishmaniases present a broad spectrum of clinical manifestations, ranging from self-healing localized or multiple cutaneous lesions to mucosal lesions and potentially fatal visceral forms. These different forms are often associated with a particular species or subgenus, nonetheless, they are not unique to a species [10]. In most cases, cutaneous leishmaniasis (CL) skin lesions are self-healing and leave permanent scars. However, some species can lead to more severe pathologies such as mucocutaneous (MCL), diffuse (DCL), or disseminated (DL) cutaneous leishmaniases. Visceral leishmaniasis (VL), also known as *kala-azar*, is the most severe form of leishmaniases and can be fatal unless treated. Common clinical signs include non-tender splenomegaly, with or without hepatomegaly, and individuals with pre-existing health conditions may develop post-kala-azar dermal leishmaniasis (PKDL) consequent to the treatment [1].

Due to the complex relationship between human, animal hosts, parasites, and sand fly vectors, the transmission of *Leishmania* spp. is intricate. Moreover, vector-borne diseases are influenced by environmental changes and socioeconomic factors such as poor housing and sanitary conditions, malnutrition, or population movement. Anthropogenic factors tend to reorient the composition and behavior of sand fly vectors. To date, there are at least 50 different sand fly species known to transmit leishmaniases. In general, each sand fly species has its preferred ecological niche and transmits a certain *Leishmania* species (reviewed in [11]). Furthermore, zoonotic leishmaniases have a broad mammalian reservoir diversity in different parts of the world [12]. The sylvatic transmission is affected by the wildlife population in and around human settlements. Divergent species of sylvatic, domestic, and synanthropic animals have been reported as reservoir hosts for various *Leishmania* species around the globe—rodents, foxes, dogs, cats, primates, hyraxes, and bats are among those maintaining the transmission of *Leishmania* [1] (Figure 1). *Leishmania* species may infect a distinct mammalian host, yet, in the northeast region of Brazil, a mosaic of different sylvatic and synanthropic rodents appear to be reservoirs of *L. (V.) braziliensis* [12]. Events such as deforestation due to urbanization can create new breeding habitats for vectors, which can lead to spillovers across ecosystem boundaries [13,14]. With over 60% of human infectious diseases being zoonotic [15], recognizing the interdependence and connections between humans, animals, and the environment that the hosts and vectors inhabit is indisputably essential. Hence, adopting a ‘One Health’ approach becomes imperative to control leishmaniases.

**Figure 2:**
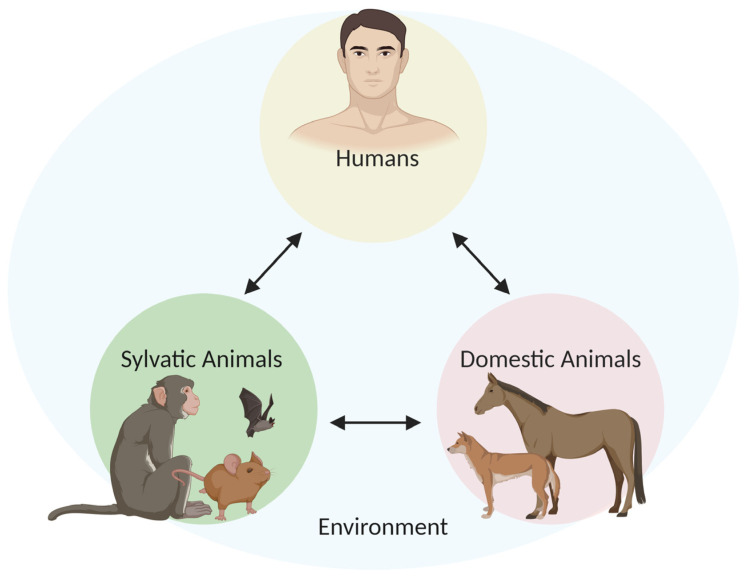
The One Health diagram illustrating the interactions between humans, sylvatic, and domestic animals within a shared environment.

The One Health approach is a global strategy for advocating multi-sectoral and trans-disciplinary collaborations in all aspects of human, animal, and environmental health while recognizing their interconnectedness [16,17] (Figure 2). The phenomenon of emerging and re-emerging infectious diseases is driven by various, often inadvertently, anthropogenic constituents, such as environmental factors (i.e., climate change, deforestation), population movement (i.e., migration, increased international travel), socioeconomic and political-driven factors (i.e., poverty, lack of political will, war/conflict), and genetic factors including host adaptation and susceptibility to infection [18]. This multiplicity of components driving disease emergence was first described by The National Academy of Sciences as a “convergence model” [19] and later defined as follows: “ecological instabilities arise from the way we alter the physical and biological environment, the microbial and animal tenants (humans included) of these environments, and our interactions (including hygienic and therapeutic interventions) with the parasites” [20].

Thus, this review aims to explore the complexities of *Leishmania* transmission and to provide an overview of various diagnostic methods and their uses in epidemiological studies to support leishmaniases control.

## 2. Clinical Manifestations

### 2.1. Cutaneous/Mucocutaneous Leishmaniases

Cutaneous leishmaniasis (CL) is endemic in over 90 countries worldwide, with approximately 0.7 to 1.2 million cases occurring every year. Yet 70–75% of cases emerge in just nine countries: Afghanistan, Algeria, Brazil, Colombia, Iran, Pakistan, Peru, Saudi Arabia, and Syria [2]. CL is caused by multiple and phylogenetically distinct *Leishmania* species, such as *L.* (*L*.) *infantum, L.* (*L*.) *tropica, L.* (*L*.) *major,* and *L.* (*L*.) *aethiopica*, which are endemic in Eurasia, and species, such as *L.* (*L*.) *amazonensis, L.* (*L*.) *mexicana, L.* (*V.*) *braziliensis,* or *L.* (*V.*) *guyanensis*, which are endemic in the Americas [1]. CL caused by *L.* (*L*.) *donovani* is unusual but some cases do occasionally arise in endemic countries such as in the southwest region of India and the northwest region of Yemen, where the majority of CL cases are caused by *L.* (*L*.) *tropica* [21,22,23]. Moreover, several atypical cases caused by *L.* (*L*.) *infantum* have been reported in the Mediterranean Basin and by *L.* (*L*.) *infantum chagasi* in Central and South American countries [24,25,26,27]. The majority of species predominantly associated with CL in humans are zoonotic, with the exception of *L.* (*L*.) *tropica* [1]. 

The clinical manifestations of leishmaniases depend not only on the parasite species but also on the host’s immune response, directing macrophages polarization towards the M1 classically activated or M2 alternatively activated phenotypes [28,29,30,31,32]. CL, particularly, can be characterized by diverse forms including localized, mucocutaneous, diffused, and disseminated forms. A large proportion of these infections are asymptomatic and/or generate minimal or no pain. However, recovered patients often experience substantial trauma and social stigmatization [33]. Localized CL lesions vary in the severity and timing of the clinical presentation and prognosis. Small erythema appears on the site of inoculation, which then develops into a papule and ulcerates over several weeks to months. Lesions caused by *L.* (*L*.) *major, L.* (*L*.) *mexicana, L. (V.) braziliensis, L. (V.) panamensis, L. (V.) guyanensis,* and *L. (V.) peruviana* often self-heal within two to eight months, whereas lesions caused by *L.* (*L*.) *infantum* and *L. (L.) tropica* tend to begin healing spontaneously after one year of disease onset, and up to five years for *L.* (*L*.) *aethiopica* [1,34].

Diffuse (anergic) cutaneous leishmaniasis (DCL) is characterized by multiple non-tender, non-ulcerated skin lesions, predominantly nodular, that contain large numbers of parasites, which may resemble lepromatous leprosy. The skin lesions typically manifest at the primary infection site, and mucosal involvement is rare, as less than five percent of these patients develop mucosal lesions [35]. *L.* (*L*.) *aethiopica, L.* (*L*.) *mexicana,* and *L.* (*L*.) *amazonensis* are responsible for the majority of DCL although other *Leishmania* species can cause this diffused form of the disease in Human Immunodeficiency Virus (HIV)-coinfected patients and present atypical manifestations such as ulceration [36,37]. Multiple lesion CL generally emerges after initially successful treatment but relapses and becomes resistant to further treatment [1,35,38]. Disseminated cutaneous leishmaniasis (DL) is largely associated with *L.* (*V.*) *braziliensis*, *L.* (*V.*) *panamenensis, L.* (*V.*) *guyanensis*, and *L.* (*L*.) *amazonensis*, and occurs mainly in Central and South America (Table 1). The disease is distinguished by multiple ulcerated papules and acneiform lesions which appear in a different site from the primary foci, and it usually transpires when the initial lesions begin to develop [35]. Patients who have recovered from *L. (L.) tropica* infections may develop a chronic form of anthroponotic CL called leishmaniasis recidivans, also known as tuberculoid leishmaniasis due to its clinical resemblance to cutaneous tuberculosis [39]. 

Mucocutaneous leishmaniasis (MCL) is a secondary stage of CL, in which 1–20% of cutaneous lesions may develop into mucosal lesions [40,41,42,43]. Nearly 90% of MCL occur in Bolivia, Brazil, and Peru [44]. Risk factors include male, older than 22 years, duration of CL, site of primary skin lesion above the waistline, absence, or delayed treatment of the primary lesions [1,45]. MCL is associated principally with *L. (V.) braziliensis* and to a lesser degree with *L.* (*V.*) *guyanensis* and *L.* (*V.*) *panamensis*. The disease may manifests itself when the parasites metastasize to the mucosal tissues of the aerodigestive tract via the lymphatic or bloodstream [1]. The clinical signs range from mild edema of the nose, upper lip, palate, and frequent local lymphadenopathy to severe mutilation with obstruction and/or destruction of the nose, pharynx, and larynx. MCL does not heal spontaneously and is potentially life-threatening with secondary bacterial infections including pneumonia and tuberculosis [34,38]. 

### 2.2. Visceral Leishmaniasis

Visceral leishmaniasis (VL) is caused by *L. (L.) donovani*, *L. (L.) infantum*, and *L. (L.) infantum chagasi* [1] (Table 1). It is noteworthy that the nomenclature of the etiological agent of VL in the Americas as a result of a decade-long discussion between experts regarding its origin [46,47]. Some suggest that *L. (L.) chagasi* is synonymous with *L. (L.) infantum*, and was introduced in the Americas during Spanish and Portuguese colonization [48,49], while some argue that *L. (L.) chagasi* existed in the Americas before colonization [50]. Regardless, *L. (L.) infantum chagasi* is the most widely accepted name for the causal species of VL in the Americas today [46].

Despite nearly 70 countries around the world being endemic for VL, over 90% of the new cases occur in seven countries: Brazil, Ethiopia, India, Kenya, Somalia, South Sudan, and Sudan [44]. It has been estimated that VL results in 0.2 to 0.4 million cases each year with a fatality rate of 10–20% when the affected individuals have access to treatment [2]. In endemic areas of East Africa and the Indian subcontinent, *L. (L.) donovani* infections are predominantly anthroponoses, whereas, across Europe, North Africa, and the Americas, *L. (L.) infantum* and *L. (L.) infantum chagasi* [1,51] are zoonoses. Moreover, there are a few records [52,53,54,55] of atypical visceral infections caused by *L. (L.) tropica* that are generally associated with CL. The clinical manifestations of VL range from asymptomatic infection to severe systemic cases associated with fever, fatigue, weight loss, pancytopenia, and non-tender splenomegaly, with or without hepatomegaly [13,56]. Hyperpigmentation of the patient’s skin can also be observed in the Indian subcontinent; hence, the disease is also called ‘kala-azar’, meaning ‘black-fever’ in Hindi [57]. 

In southern Europe, up to 70% of the cases in adults are linked to HIV infection. It has been shown that HIV infection increases the risk of developing VL by 100 to 2320 times [58]. Moreover, HIV-coinfected patients may have an impact on disease transmission as several studies have demonstrated that these patients are highly infectious to sandflies [1]. Furthermore, coinfection with VL can lead to faster progression to Acquired Immune Deficiency Syndrome (AIDS) [58]. HIV coinfection can also often induce atypical leishmaniases, for instance, infections with *L. (L.) amazonensis* [59] and *L. (V.) braziliensis* [58] can cause VL. Several reported cases in endemic countries show VL can be transmitted through a non-vector transmission route, such as blood transfusion [60,61] or via the sharing of needles between intravenous drug users [62]. Congenital VL cases, although extremely rare in humans, were found to be the predominant route of transmission among canines in the U.S. [63], suggesting *Leishmania*’s potential in invading the placenta [64] (reviewed in [65]).

### 2.3. Post-Kala-Azar Dermal Leishmaniasis

Post-kala-azar dermal leishmaniasis (PKDL) is a complication that arises from 6 to 12 months after the recovery of VL. It occurs in all areas endemic for *L. (L.) donovani*, particularly in East Africa and the Indian subcontinent, which are responsible for up to 50% and 10–20% of the estimated VL cases, respectively [66]. However, a few cases caused by *L. (L.) infantum* in immunosuppressed individuals were reported, with one case implying that highly active antiretroviral treatment (HAART) may lead to the development of PKDL [67,68]. Clinical manifestations of PKDL vary due to one’s immune responses but generally can be described as one of the three main manifestations: hypopigmented macules, malar rash on the face, and nodular lesions [69,70]. Yet, rare signs of the disease have been reported from endemic regions, including oral and genital mucosa lesions [69]. Macules and lesions from these patients often contain a few parasites that seem to persist in the skin after treatment [71]. Having said that, those individuals are important reservoirs for anthroponotic VL, although PKDL is not fatal [72]. A recent xenodiagnoses study from the Indian subcontinent emphasized the significance of PKDL and its contribution to the ongoing transmission of *L. (L.) donovani.* Three patients with PKDL, maculopapular and/or nodular forms, were infectious to sand fly vectors [73].

### 2.4. Asymptomatic Infection

Asymptomatic infections of both VL and CL (reviewed in [74]) are common in endemic countries. Although it has not yet been proven that human asymptomatic carriers can transmit *Leishmania*, asymptomatic canines have been shown to infect sandflies at lower rates—18.3% versus 51.9% for symptomatic canines [75]. Thus, human asymptomatic cases may have the potential to alter the transmission dynamics, making it harder to estimate the global burden of the disease [76]. Several studies have recorded the ratio of asymptomatic to symptomatic infections of VL in endemic areas: 4:1 in Kenya [77], 7.9:1 to 8.9:1 in India and Nepal [78,79], 6.5:1 to 89:1 in Brazil [13,80,81], and 13:1 in Iran [82]. A recent active mass survey from a highly endemic district for VL in India reported that, among individuals who tested positive, 42% of them were asymptomatic cases, and nearly 10% of the asymptomatic cases developed symptomatic VL during 36 months of follow-up [78]. Moreover, within the same district, researchers found that two percent of the patients who developed PKDL had no known history of VL, suggesting that asymptomatic cases of VL may develop into PKDL [72]. Additionally, a cohort study conducted after the leishmaniasis outbreak in Madrid (Spain) in 2009 observed the prevalence rate of asymptomatic infection to be nearly 20%. Moreover, 38.3% of the participants reported that they had close contact with dogs, and 24.6% of the 38.3% of participants were asymptomatic carriers. As domestic dogs are the most common reservoir host of *L. (L.) infantum* in Spain, these findings suggest that having frequent contact with dogs may increase the likelihood of becoming an asymptomatic carrier of VL [75]. 

Asymptomatic carriers can also transmit *Leishmania* through blood transfusions. Nearly 10% and 5% of the blood donors from Granada (Spain) and Salvador (Brazil), respectively, tested positive for asymptomatic *Leishmania* infection, raising concerns about the safety of the blood supplies in endemic regions [60,83].

## 3. Risk Factors for Leishmaniases

### 3.1. Socioeconomic Factors and Malnutrition

Although leishmaniases are widespread across continents, the risk of these diseases is much greater for those living in poverty. Treatment for leishmaniases is comparatively more expensive than other poverty-related diseases. Malaria treatment, for instance, costs between USD 0.10 and USD 2.40 per course [84], whereas, for leishmaniasis, the drug alone typically costs between USD 30 and USD 1500 [85]. In Nepal, the average cost of anti-leishmanial drugs is greater than the median annual household per capita income, and many households opt to sell their livestock to cover the treatment cost, leading to a devastating financial impact for affected families [86]. Moreover, many of those in poverty cannot afford the cost of prevention. A survey report from Afghanistan revealed that 78% of respondents said they cannot afford a bed net [87]. 

Undeniably, poor living conditions are often associated with a higher risk of *Leishmania* infection [88]. A recent study in Nepal where the researchers examined housing structures and land lots in endemic districts found that houses with natural floors increased the risk of infection by eightfold, walls made from straw, leaves, and/or bamboos increased by threefold, walls with cracks, especially in the bedroom, increased by threefold and proximity to a livestock shed, particularly located in front or at the side of a dwelling, was shown to increase the risk by fourfold [89]. Furthermore, living in overcrowded homes may also increase the risk of infection since anthropophilic sand fly vectors are attracted by human kairomones and carbon dioxide, which results in increased density of sand fly vectors in these homes [90,91]. Likewise, a study from rural Bangladesh reported that living with a recent anthroponotic VL patient increases the risk of infection by 26-fold [92]. While overcrowded households may have a direct influence on their increased risk, there are other shared risk factors such as malnourished households that contribute to the likelihood of becoming infected [92]. 

Malnutrition increases the host susceptibility and is also a determinant for the severity and clinical manifestations of the disease [1]. An in vivo study with mice fed on altered protein, iron, zinc, and calorie intakes indicated that malnutrition can cause a failure of the lymph node barrier function after infection with *L. (L.) donovani*, and promote early visceralization [88]. These findings are supported by field observations in Ethiopia that showed malnourished individuals were three times more susceptible to developing VL [93]. Similar cases were also seen in animals; dogs with poor nutritional status were about 13 times more susceptible to *L. (L.) donovani* infection than those in good health [94]. Furthermore, malnutrition has also been associated with an increased risk of various complications of leishmaniases including MCL and PKDL [1]. 

### 3.2. Migration

Anthroponotic leishmaniases occur predominantly in rural and urban areas, and the diseases are usually characterized by large outbreaks in densely populated cities, especially in war and conflict zones, refugee camps, and in settings where there is large-scale population migration [1]. Several studies revealed a strong relationship between civil unrest and VL epidemics. For instance, one of the most devastating VL outbreaks occurred during 22 years of civil war in South Sudan, forcing the exodus of more than four million people (reviewed in [95]). A community-based longitudinal study in South Sudan divulged a significantly higher leishmaniases mortality rate in areas of civil war, unrest, and human displacement [2]. Furthermore, due to the sudden influx of displaced refugees, the number of VL cases reported in neighboring Eastern African countries doubled. More than 400,000 South Sudanese refugees fled to the woodland region of the state of White Nile in Sudan, and another 300,000 refugees migrated along the Omo Valley and Gambela regions in Ethiopia. These migrations resulted in the re-emergence of the VL epidemic in those two regions [95]. A similar trend was also observed in Lebanon between 2001 and 2014 due to the Syrian conflict and the subsequent influx of refugees. In previous years, the average number of reported leishmaniases cases was zero to six per year, whereas in 2013, more than 1000 new cases were reported [96].

The rapid development of road transportation systems encourages the migration of people and their *Leishmania*-infected dogs to non-endemic regions. In Brazil, 95.4% of zoonotic VL cases were restricted to the north and northeast regions of the country until 2003. The major road infrastructure development across the country resulted in a mass migration of people seeking better job opportunities in the central, south, and southeast regions of Brazil. The cases reported in north and northeast regions decreased to 78.1% while in the central and southeast regions of the country, observed cases increased to 21.8% [97,98]. In the state of São Paulo, located in the southeast region of Brazil, there was a gradual increase in human VL along the Marechal Rondon highway, Novoeste railway, and the Bolivia–Brazil gas pipeline [98]. It is noteworthy that replacing the animal agriculture with sugar cane plantations in São Paulo may have contributed to the spread of the disease, as harvesting the cane involves migrant labor, often from the northeast region [99].

### 3.3. Environmental Changes

Changes in the environment also have a strong impact on leishmaniases, either due to climate change caused by global warming or man-made ecological alterations. An abrupt climate change exerts influence on the migration of sand fly vectors and reservoir animals. Numerous studies have shown that leishmaniases disease distribution has been shifting and will shift as a response to climate change [100]. Moreover, population growth appears to be the significant driver of clearing forests due to the increased demand for agricultural products and the expansion of transmigration settlements [101]. It is indisputable that widespread deforestation has led to an expeditious increase in leishmaniases cases, and peri-domestic, peri-urban, and urban transmission [1]. As such, the number of annual cases of CL in Costa Rica has increased by 46% from 2002 to 2007 [2], and the incidence rate of CL has increased by 30% from 1998 to 2002 in Brazil [102], with both cases assumed to be associated with the result of deforestation. The transmission rate for zoonotic leishmaniases is the highest among the marginalized population and small frontier farmers living at the edge of natural foci nearby human dwellings and wild habitats [1,103]. For instance, the city of Manaus in the state of Amazonas (Brazil) is the largest urban settlement within the Amazon rainforest, with a population of over 1.7 million. In Amazonas, where more than 40% of the leishmaniases cases occur in Brazil, *L.* (*V*.) *guyanensis* is the prevalent species [104]. Due to the rapid urbanization and expansion of the city and suburbs on the edge of the primary forest, researchers suspect that the sylvatic transmission of *L.* (*V*.) *guyanensis* had spilled over into a peri-domestic transmission cycle, and eventually reached the urban transmission cycle [100]. 

The relationship between agricultural expansion and leishmaniases seems evident, as the abundance of sand fly vectors and increased exposure to infection with *Leishmania* have been recorded in several coffee plantations in Brazil, Colombia, and Mexico [105,106,107]. Moreover, the development of new irrigation technologies appears to promote environmental changes, resulting in the attraction of certain sand fly species from neighboring regions/countries. In Central Tunisia, where *Phlebotomus perniciousus* and *P. papatasi* used to be the principal vectors of *L. (L.) infantum*, *P. perfiliewi* became the most abundant sand fly species in irrigated areas. Reportedly, the overall abundance of *P. perfiliewi* in Central Tunisia was at 5% prior to irrigation development [108,109]. 

## 4. Diagnostic Tools for Leishmaniases

**Figure 3:**
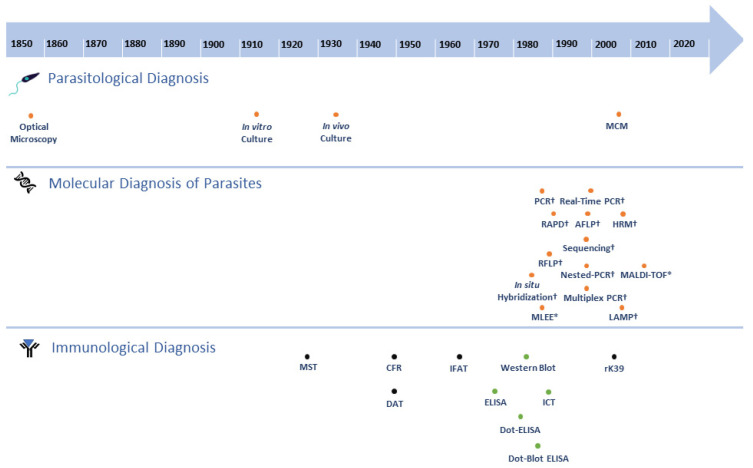
Timeline of the development of diagnostic tests for leishmaniases. Orange dot, direct method; black dot, indirect method; green dot, direct and/or indirect method; *, protein-based molecular method; †, nucleic acid-based molecular method; Parasitological Diagnosis: 1885, Optical Microscopy [111,112,113]; 1912, in vitro culture [114]; 1935, in vivo culture [115]; 2004, MCM (Microcapillary Culture Method) [116]. Molecular Diagnosis of Parasites: 1987, in situ Hybridization [117]; 1990, PCR (Polymerase Chain Reaction) [118]; 1990, MLEE (Multilocus Enzyme Electrophoresis) [49]; 1991, RFLP [119]; 1993, RAPD (Random Amplification of Polymorphic DNA) [120]; 1997, AFLP (Amplified Fragment Length Polymorphism) [121]; 1998, Nested-PCR [122]; 1998, Multiplex PCR [123,124]; 1999, Sequencing [125]; 2001, Real-Time PCR [126]; 2009, LAMP (Loop-mediated Isothermal Amplification) [127]; 2010, HRM (High Resolution Melting) [8,128]; 2014, MALDI-TOF (Matrix-Assisted Laser Desorption Ionization-Time-of-Flight Mass Spectrometry) [129]. Immunological Diagnosis: 1926, MST(Montenegro Skin Test) [130]; 1947, CFR (Complement Fixation Reaction) [131]; 1947, DAT (Direct Agglutination Test) [132]; 1964, IFAT (Indirect Immunofluorescence Antibody Test) [133,134]; 1978, ELISA (Enzyme-Linked Immunosorbent Assay) [135]; 1983, Dot-ELISA [136]; 1984, Western Blot [137]; 1987, Dot-Blot-ELISA [138]; 1990, ICT (Immunochromatographic Test) [139]; 2000, rK39 (rapid anti-K29 antibody Immunochromatographic strip-test) [140].

Despite the recent advances in diagnostic tools, diagnosing leishmaniases still imposes substantial challenges in the remote areas of endemic countries around the globe. Moreover, due to its complex transmission cycle, involving the various biological entities, identifying the responsible *Leishmania* species is crucial in disease control and interventions [1]. Early and accurate detection as well as improving patients’ outcomes [110] also provides imperative data for eco-epidemiological studies to monitor and assess the outbreak and evaluate current control measures that are in place in endemic regions. A diagnosis of leishmaniases is often made by evaluating the clinical manifestations of the disease in the patient, followed by one or more laboratory diagnostic tests (Figure 3).

### 4.1. Parasitological Diagnoses

Parasitological diagnoses employ tissue aspirates from the spleen, bone marrow, lymph nodes, or peripheral blood of suspected individuals with VL, or skin biopsies/smears from ulcers/lesions of suspected individuals with CL or PKDL [1]. The presence of the parasites in samples can either be directly visualized by optical microscopy or cultured in appropriate culture media for later microscopic visualization (in vitro culture) [1]. The viability of the parasites present in the tissue samples can also be assessed by inoculation into susceptible animals followed by infectivity analysis (in vivo culture) [115]. Microscopic examination provides high genus specificity, although its sensitivity can vary depending on the different tissue aspirates. It also presents a relatively high risk of contamination and requires experienced technicians when performing the examination [66,141]. Parasite culturing is not as routinely used in a clinical setting as microscopic examination, yet culturing can enhance the detection sensitivity and isolated parasites can then be identified to the genotype and species level [34]. Among the various culture media to cultivate desired parasites, Solid Novy–MacNeal–Nicolle (NNN) medium is the most widely used for isolating and culturing the parasites although improvements were found using a more nutritious media [142]. However, in recent decades, a Microcapillary Culture Method (MCM) surfaced to increase the sensitivity of parasite culturing techniques by using peripheral blood mononuclear cells and buffy coats. Noteworthily, MCM only requires a small volume of culture medium, and it is cheaper than other media [143]. 

### 4.2. Molecular Diagnosis of Parasites

The identification of *Leishmania* species by various methods exploring specific characteristics of nucleic acids has been described in recent decades. One of the earliest applications of molecular diagnosis for discriminating *Leishmania* species employed Restriction Fragment Length Polymorphism (RFLP). Researchers used P32-labelled radioactive kDNA probes to hybridize the digested fragments of DNA by Southern blotting [119]. Shortly after, the in situ hybridization technique was described for the detection of *Leishmania* in blood and smear samples from in vivo studies using non-radioactive DNA probes [117]. However, molecular diagnoses then were not widely implemented in clinical settings due to the complexities, cost, and time requirements, until the Polymerase Chain Reaction (PCR) was developed [144]. The detection of parasites’ nucleic acids by PCR has not only accelerated the confirmation of the diagnosis but also constituted a cogent tool to enhance control and surveillance of leishmaniases by identifying causative species [145], sylvatic origins [12,146], and their geographical distribution. The advancement of PCR diversified diagnostic assays targeting regions of the parasites’ DNA in the mitochondrial genome (e.g., kinetoplast DNA or kDNA) [147,148], nuclear genome (e.g., SSU rDNA [149,150], *glucose-6-phosphate dehydrogenase* (*g6pd*) [151], *70kDa heat-shock protein* (*hsp70*) [152,153], *amino acid permease 3* (*aap3*) [154], *cysteine proteinase B* (*cpb*) [155], or intergenic genomic regions (ITSs) [156]). The main advantage of the PCR method is its high sensitivity and specificity. On the other hand, the limitations of this method are the relatively higher risk of contamination and its restricted detection range, which may result in variations in the method’s sensitivity [157,158].

Subsequently, numerous PCR-based diagnostic methods were developed to further improve the detection sensitivity [159]. These methods include DNA hybridization coupled with conventional PCR, Randomly Amplified Polymorphic DNA (RAPD), and Amplified Fragment Length Polymorphism (AFLP) [160,161]. However, these methods also present some limitations. For instance, RAPD has poor reproducibility and requires the laborious standardization of PCR conditions [162,163,164].

Standard PCR protocols were also modified to achieve improved outcomes, such as the nested PCR approach. This approach was first used in the late 1990s to distinguish the Eurasia species *L.* (*L*.) *tropica*, *L.* (*L*.) *infantum,* and *L.* (*L*.) *major* based on their amplicon sizes using kDNA as a target [122]. In another study, researchers designed specific primer sets targeting G6PD to distinguish between the two subgenera of *L. (Leishmania)* spp. and *L. (Viannia)* spp., then *L.* (*V*.) *braziliensis* from other *L. (Viannia)* spp. [151]. It is noteworthy that the discrimination of *L.* (*V*.) *braziliensis* from the subgenus *L. (Viannia)* is important for disease prognosis because infections by *L.* (*V*.) *braziliensis* often lead to MCL [151]. The main advantage of nested PCR is its higher accuracy, sensitivity, and specificity compared to conventional PCR. Nevertheless, a higher risk of contamination can be a major disadvantage of this method [143]. Additionally, the multiplex PCR approach/technique was developed. The application of multiplex PCR is particularly useful for rapid in-country identification of *Leishmania* parasites in endemic areas where more than one species resides. For instance, researchers developed a one-step differentiation for the American species of *L.* (*V*.) *braziliensis*, *L.* (*L*.) *mexicana*, and *L.* (*L*.) *donovani,* while minimizing the time and resources. As the multiplex PCR method allows for the detection of multiple targets simultaneously, the main advantages of the method are cost and time effectiveness. However, the standardization of PCR conditions can be laborious and this method is relatively less sensitive that other PCR-based methods [123].

On the other hand, a loop-mediated isothermal amplification (LAMP) flourished as an alternative technique for other PCR-based diagnostic tools. It is a species-specific DNA amplification method comparable to other PCR-based methods. Unlike PCR, in which the amplification is carried out with repeated thermal cycling, isothermal amplification is carried out at a constant temperature without needing a thermal cycler, which can be advantageous for field use [165]. In addition, compared to other PCR-based methods, LAMP has shorter reaction times [166]. However, the biggest drawback of this technique is that the amplified products cannot be used for downstream applications. Moreover, depending on the sample used, this technique can be less sensitive than conventional PCR (reviewed in [165]).

Sequencing became indispensable not only for the precise identification of *Leishmania* species [167] but also to discriminate *Leishmania* from other trypanosomatids. In the state of Mato Grosso do Sul (Brazil), naturally occurring co-infections of *L.* (*L*.) *infantum chagasi* and *Trypanosoma evansi* in dogs were identified by PCR associated with sequencing of target SSU rDNA [168]. Sequencing is also efficacious in distinguishing *Leishmania*-like parasites presenting leishmaniases-like disease [169]. Detecting *Leishmania*-like parasites is extremely challenging as these organisms morphologically resemble *Leishmania* under a microscope and show a positive reaction to the rK39 test. Moreover, these *Leishmania*-like parasites’ DNA can also be amplified by several commonly used primers targeting different genomic regions of *Leishmania*. In a recent study, a group from Brazil identified a *Crithidia*-related, non-*Leishmania* parasite from a patient with the VL-like disease by whole-genome sequencing, underlining the need for the accurate identification of *Leishmania*-like parasites [169]. Further difficulties were also encountered when detecting species of the subgenus *L.* (*Mundinia*) in patients. It was initially thought that isolates of *L. (M.) martiniquensis* were monoxenous trypanosomatids [170], until it was later discovered that they too belonged to the genus *Leishmania.*

The use of Real-Time PCR emerged as an advanced PCR-based method in the early 2000s and was first described for the quantification of parasite load in the liver of *L.* (*L*.) *infantum*-infected mice [126]. Real-Time PCR allows the continuous monitoring of the synthesized PCR products [143,171]. In a study, researchers undertook Real-Time PCR tests targeting *g6pd* to identify and quantify the responsible species for the American CL [172]. Similarly, another study was able to distinguish nine different species from both *L. (Leishmania)* and *L. (Viannia)* subgenera and to quantify parasite load for in vivo studies with Real-Time PCR targeting *aap3* coupled with a multiplex strategy [154]. The main advantages of Real-Time PCR are its increased sensitivity and reduced contamination risk, as the method does not require post-amplification handling, besides the possibility of adapting it to a high throughput format (reviewed in [171]). However, some limitations are the requirement of expensive and specialized equipment, and experienced personnel to analyze the results [158].

High-resolution melting (HRM) analysis is one of the most recent developments in molecular diagnosis as a variation on Real-Time PCR. This is a robust technique for the detection of polymorphisms, mutations, and epigenetic differences in DNA samples by monitoring dissociation kinetics of PCR products (reviewed in [173]). While the use of HRM analysis for *Leishmania* spp. is still infrequently described in the literature, this method has been applied in identification protocols with results that vary in terms of approach and its discriminatory capacity. The use of an HRM-like approach to discriminate the Eurasian species *L. (L.) infantum*, *L. (L.) donovani*, *L. (L.) tropica*, and *L. (L.) major* by targeting their kDNA polymorphisms was first described in the early 2000s [174]. Subsequently, the use of kDNA enabled the discrimination of the subgenera *L. (Leishmania)*, *L. (Viannia)*, and the species *L. (L.) amazonensis* from *L. (L.) infantum* in isolated strains and clinical samples [175,176]. Some recent studies targeting ITS-1 rRNA and *hsp70* allowed the discrimination of six different American *Leishmania* species, *L. (L.) infantum chagasi, L. (L.) amazonensis, L. (L.) mexicana, L. (V.) braziliensis, L. (V.) guyanensis,* and *L. (V.) panamensis* [177]. Moreover, the melting and dissociation profiles of three different regions in *hsp70* or *aap3* enabled the differentiation of the main causative species of leishmaniases in Africa, Eurasia, and the Americas [178,179]. In addition to the advantages of Real-Time PCR, HRM provides even higher specificity than using Real-Time PCR alone, as the method allows better discrimination of genetic variants [173].

The analysis of electrophoretic profiles of isoenzymes, or Multilocus Enzyme Electrophoresis (MLEE), is considered as a gold standard technique by the WHO [1]. This method is based on the differences in the electrophoretic profiles of several enzymes obtained from promastigotes grown in culture, which can generate highly informative profiles regarding the identity of the tested organisms. This technique made significant contributions to the taxonomy of the genus *Leishmania* and discriminated between Eurasian and American species [143], yet it is not suitable for routine diagnosis, since it depends on the establishment of a culture of parasites and the requirement of specialized equipment [156,180].

Lastly, Matrix-Assisted Laser Desorption Ionization-Time-of-Flight (MALDI-TOF) mass spectrometry (MS) has emerged in recent years as a robust tool for the identification of microorganisms. The protein spectra, or “fingerprint”, of an isolate are compared to a reference spectral database, definitively identifying the isolates within an hour [129]. This technique, also known as MALDI BioTyper Systems, has been successfully applied to various pathogens in clinical laboratory settings [181]. Several studies have demonstrated the use of MALDI-TOF MS to accurately discriminate *Leishmania* spp. [129,182]. Nevertheless, like MLEE, this technique requires the isolation and cultivation of parasites and, due to the high cost of equipment and maintenance, it is currently only available in larger research laboratories or reference centers [129].

### 4.3. Immunological Diagnoses

Immunological diagnoses are based on either the presence of host-specific antibodies (indirect) and/or the detection of the parasite’s antigens (direct) (reviewed in [172]) (Figure 3). Methods include the Montenegro Skin Test (MST), also called the *Leishmanin* Skin Test (LST), Complement Fixation Reaction (CFR), Direct Agglutination Test (DAT), Indirect Immunofluorescence Antibody Test (IFAT), various forms of Enzyme-Linked Immunosorbent Assay (ELISA), Western blotting, Immunochromatographic Test (ICT), and the rK39 antigen-based immunochromatographic test. Immunological diagnoses show comparatively good diagnostic accuracy, especially during the acute stage of VL. On the contrary, they are not widely used for CL due to their low sensitivity and variable specificity, as cutaneous lesions often show lower levels of antibodies [1,183]. This could be since antibodies are partly species specific, and if a heterologous antigen is used, its detection may be low or undetectable [184]. Nonetheless, in the diagnosis of CL, Dot-Blot [138], and IFAT [185] have demonstrated high sensitivity. The Montenegro skin test is the most frequently used method for CL as it can detect the occurrence of a delayed-type hypersensitivity response [1,143]. In the diagnosis of VL, ELISA is the most widely used and sensitive immunological diagnostic method. In a study comparing sensitivities of standard ELISA, Dot-ELISA, and CFR to detect *L. (L.) donovani*, Dot-ELISA showed the highest sensitivity of 89%, followed by the standard ELISA and CFR, 80% and 72%, respectively [136]. Irrespective of the method, the sensitivity of serological tests depends on the antigens used [186]. For instance, when a study evaluated the sensitivity and specificity of the recombinant proteins rK9, rK26, rK39, and Crude Soluble Antigen (CSA), researchers found sensitivities of 78%, 38%, 100%, and 80% and specificities of 84%, 80%, 96%, and 72%, respectively [183,187]. Furthermore, rK39 was developed for field use, and it is recognized for its low-cost, quick and easy procedures, and relatively high reproductivity [1,71]. Nevertheless, the biggest drawback of utilizing any of these immunological tests is that they are unreliable for the diagnosis of relapses as specific antibodies can remain detectable up to several years post-recovery [1], and they are not species specific.

## 5. Use of Diagnostic Tools in Epidemiological Studies

Diagnostic tools have many important applications in population-based studies and disease surveillance for assessing the endemicity in specific areas, estimating the prevalence, and other various epidemiologic studies such as quantitative risk assessment [188].

Disease surveillance is critical in monitoring the disease burden, providing early warnings of an outbreak, determining risk factors, and evaluating the effectiveness of implemented control measures [189]. Active sampling is required for surveillance, followed by laboratory tests to improve the accuracy of the system. From 2008 to 2014 in northern Spain, researchers collected spleen samples from deceased wolves and small carnivores to test for *L. (L.) infantum* DNA using conventional PCR. The study, which was a part of the wildlife sanitary surveillance programs, not only revealed a widespread presence of the parasite in previously evaluated as non-endemic areas but also promoted the use of the wolf as a sentinel for leishmaniases [190]. Moreover, geographical information system (GIS) techniques can be applied to enhance surveillance systems and identify their causes. GIS integrates a broad range of information from different sources including diagnostic results and environmental data, to analyze and display the geographically referenced or spatial distribution of the parasite, host, or vector, hence enabling the identification of disease occurrence patterns and probable risk factors [191]. In the Central Western regions of São Paulo (Brazil), road-killed wild animals were subjected as sentinels for detecting *L. (L.) infantum chagasi* DNA using PCR. It is noteworthy that monitoring road-killed wild animals not only provides insights about their health and the environment but also about their behaviors as, often, their natural habitat is disrupted by road construction. The geographic coordinates of the infected animals were recorded by using GIS, which gave rise to a better understanding of pathogen distribution [192]. In another study from Nepal, researchers combined DAT results with GIS to evaluate the exposure of human and domestic animals to *L. (L.) donovani* and showed the occurrence of spatial clustering of seropositive humans and domestic animals [6]. Further to this, several studies in endemic countries observed a correlation between meteorological factors (e.g., temperature, precipitation, humidity) and the incidence and distribution of serological titers of the infected canines [193]. 

The disease prevalence estimate is the proportion of positive results obtained from diagnostic tests to the entire tested population, and the seroprevalence estimate of the disease serves as a proxy for the outcome of interest [188]. In a longitudinal study in the state of Minas Gerais (Brazil), researchers employed ELISA to screen and IFAT to confirm the infection, and a Dual-Path Platform (DPP) immunoassay was performed with the stored sera samples after the end of the collection period. A high prevalence of seropositive dogs was found in areas with a high density of free-roaming dogs and significantly contributed to the continuous circulation of leishmaniases among the human population [194].

To estimate the exposure effect of a response variable based on the expected risk prevalence factor and diseases, a risk prediction study can be performed with cohort, case–control, or cross-sectional study designs [188]. In west Ethiopia, a cross-sectional study was conducted to determine the seroprevalence of human VL and associated risk factors. Researchers used ITleish, a rapid immunochromatographic test, and the Montenegro skin test for the survey, along with semi-structured questionnaires to identify associated risk factors [195]. In another study, researchers performed a cohort study among U.S. soldiers who were deployed to Iraq between 2002 and 2011 to determine the prevalence and asymptomatic VL risk factors among these soldiers. Several tests including ELISA, rK39, and Real-Time PCR were used to test blood samples, and a detailed risk factor survey was conducted for the study. Nearly 20% of the soldiers tested positive for asymptomatic VL, and those who were deployed to the Ninewa governorate showed a strong association with infection [196]. Furthermore, to assess the disease risk associated with the movement of host reservoirs and/or vectors, a quantitative risk assessment can be performed [188]. In South Africa, researchers performed IFAT and Real-Time PCR for an initial screening, followed by PCR and sequencing analysis to diagnose leishmaniasis in imported dogs and evaluate the risk of introducing canine leishmaniasis in the country [197].

## 6. Conclusions

The control of leishmaniases is an ongoing global challenge complicated by many different biological and environmental factors involved in the circulation of the diseases. Furthermore, the leishmaniases are re-emerging in endemic areas and emerging in non-endemic areas due to the increasing human influence on the environment. Taking the complexity of the disease into account, integrating the One Health approach is the essential key to controlling the disease. Moreover, diagnosing the leishmaniases is arduous due to its broad spectrum of clinical manifestations that often overlap with other co-endemic infectious diseases. The rapid advancement of various diagnostic tests in recent decades has improved early detection and accurate species identification. Diagnostic tests are also pivotal in performing various epidemiological studies, including active surveillance, and identifying risk factors. Considering the relationship between zoonotic diseases and spillover events (Figure 1), the use of synanthropic animals as sentinels is vital for surveilling zoonotic leishmaniases in the human population. However, detecting zoonotic spillover events requires rapid and large-scale testing, which require special equipment and trained personnel that are often not available in the resource-poor countries most affected by the disease. Future efforts must be directed towards the development of cost-effective and more accurate, accessible tests designed for field use in remote areas of endemic regions.

## Figures and Tables

**Table 1:** Clinical manifestations, reservoir host, and geographical distribution of *Leishmania* species (modified from [1]).

	Species	Clinical Manifestation	Reservoir Host	Country/Region
Eurasia (Old World)	*L. (L.) donovani*	AVL, PKDL, CL	Human	VL: West and Central Asia, China, The Indian subcontinent, The Mediterranean Basin, East Africa; CL: The Mediterranean Basin; ML: North Africa; PKDL: The Indian subcontinent, East and North Africa
	*L. (L.) infantum*	AVL, ZVL, CL	Human, Dog, Fox, Jackal, Badger, Rodent, Cat, Opossum	VL: Central and West Asia, China, The Mediterranean Basin, Africa; CL: The Mediterranean Basin, West Asia, China, West Africa
	*L. (L.) major*	ZCL	Rodent	West and Central Asia, The Indian subcontinent, The Mediterranean Basin, Africa
	*L. (L.) tropica*	ACL, ZCLAVL	Human, HyraxHuman	Central, South and West Asia, The Mediterranean Basin, East Africa West Asia
	*L. (L.) killicki*	CL	Unknown	The Mediterranean Basin
	*L. (L.) aethiopica*	ZCL, DCL, ML	Hyrax, Rodent	CL: East Africa (Ethiopia and Kenya); ML: Ethiopia
	*L. (M.) orientalis*	CL, DL, VL	Unknown	Thailand
Americas (New World)	*L. (L.) infantum chagasi*	ZVL, CL	Dog, Cat, Fox, Opossum	South and Central America, Mexico
	*L. (L.) mexicana*	ZCL, MCL, DCL	Rodent, Opossum	Americas
	*L. (L.) pifanol*	DCL	Unknown	Venezuela
	*L. (L.) venezuelensis*	CL	Unknown	Venezuela
	*L. (L.) garnhami*	ZCL	Unknown	Central America, Venezuela
	*L. (L.) amazonensis*	ZCL, DCL, CL	Rodent	South America
	*L. (V.) braziliensis*	ZCL, MCL, DL	Dog, Horse, Donkey, Mule, Rodent, Opossum	South and Central America, Mexico
	*L. (L.) waltoni*	DCL	Unknown	Dominican Republic
	*L. (V.) guyanesis*	ZCL, MCL	Sloth, Anteater, Opossum	South America
	*L. (V.) panamensis*	ZCL, MCL	Dog, Sloth, Opossum, Tamandua	South and Central America
	*L. (V.) shawi*	ZCL	Sloth, Primate	Brazil
	*L. (V.) naiffi*	ZCL	Armadillo	Brazil, French Guiana
	*L. (V.) lainsoni*	ZCL	Rodent	South America
	*L. (V.) lindenbergi*	ZCL	Unknown	Brazil
	*L. (V.) peruviana*	ZCL, MCL	Dog, Opossum, Rodent	Peru
	*L. (M.) martiniquensis*	CL	Unknown	French Guiana
	*Endotrypanum colombiensis*	ZCL	Sloth	Colombia, Venezuela, Panama

ACL, anthroponotic cutaneous leishmaniasis; AVL, anthroponotic visceral leishmaniasis; CL, cutaneous leishmaniasis; DL, disseminated cutaneous leishmaniasis; DCL, diffuse (anergic) cutaneous leishmaniasis; MCL, mucocutaneous leishmaniasis; ML, mucosal leishmaniasis; PKDL, post-kala-azar dermal leishmaniasis; VL, visceral leishmaniasis; ZCL, zoonotic cutaneous leishmaniasis; ZVL, zoonotic visceral leishmaniasis.
